# Supplementary material for: High-dimensional phenotyping reveals novel macrophage-like and hybrid subsets within murine splenic conventional dendritic cells
Source: PLoS One. 2026 Jan 30;21(1):e0341819. doi: 10.1371/journal.pone.0341819 (PMC12857976; doi:10.1371/journal.pone.0341819)
Supplement: Attachment — Submitted filename: Response to Reviewers.docx [file pone.0341819.s002.docx]

**Response to Reviewers**

**Manuscript ID: PONE-D-25-48158**

**Title**: High-Dimensional Phenotyping Reveals Novel Macrophage-Like and Hybrid Subsets within Murine Splenic Conventional Dendritic Cells

We sincerely thank the Academic Editor and the reviewers for their constructive comments and for granting an extension to complete these revisions. We have carefully considered all points and have made extensive revisions to the manuscript. Below is our point-by-point response to all editorial and reviewer comments. All changes are highlighted in the “Revised Manuscript with Track Changes” file. A clean version of the manuscript is also provided.

**Response to Journal Requirements**

1. **Style Requirements:**

We have reformatted the manuscript to meet PLOS ONE’s style requirements, including the use of the provided templates for file naming and document structure.

2. **Funding Information:** The Funding Information and Financial Disclosure sections have been reconciled. The updated statement has been reconciled and is now included in the manuscript (placed before Data Availability Statement) is: “This work was supported by the Natural Science Foundation of Shandong Province (ZR2024MH129), the National Natural Science Foundation of China (82301629), and the Project of the Innovative Training Program for Students at Jining Medical University (cx2020118). There was no additional external funding received for this study.”
3. **Data Availability Statement:**

**A Data Availability Statement** has been added to the manuscript (placed before the References). The statement reads: “All relevant data are within the manuscript and its Supporting Information files. The minimal dataset required to replicate the results, including the values behind all means, standard deviations, and other measures reported, as well as the data points used to build graphs, are provided as Supporting Information (Mouse_FlowCytometry_CellSubsets_ThreeGroups).” We have prepared the file Mouse_FlowCytometry_CellSubsets_ThreeGroups containing the complete analytical dataset and will upload it as a **Supporting Information** file.

4. **Figure Quality:**

We have re-exported **Figures 1, 2, and 3** as high-resolution TIFF files (300 DPI, CMYK color space, LZW compression) in strict compliance with PLOS ONE’s figure guidelines.

5. **References:**

We have reviewed and formatted the entire reference list in strict accordance with PLoS ONE’s citation style guidelines. For the non-existent references (No.22 and No.24), we have removed these invalid citation marks from the manuscript following careful verification, and re-numbered all references sequentially to ensure consistency between in-text citations and the reference list. Additionally, we have added the recommended citation for flow cytometry guidelines (Cossarizza et al., 2019), which is now integrated as Reference [**19**] in the revised manuscript.

**Response to Reviewer #1**

**Comment 1:**

The work only explores the immunophenotypes of the identified cDC subsets but does not look into their functional relevance. How, then, do the authors confirm that the cDCs are indeed acting as APC? This question becomes even more relevant as the authors claim to have "functionally verified" the cDC subsets, but have only tested their sensitivity to liposomal clodronate. Liposomal clodronate does not directly guarantee their antigen presenting capacity, and is only an indicator of their phagocytic activity. Additional experiments demonstrating the antigen presentation capacity of these cDCs (like surface MHC expression, ability to trigger TCR-proximal signaling in T cells etc) must also be done if the authors prefer sticking to the statement that they have “functionally verified” the cDC subsets.

**Response:**

We agree that functional validation of antigen presentation is important. However, due to time and technical constraints within the current revision period, we were unable to perform additional functional assays such as MHC presentation or T-cell activation. Instead, we have toned down claims of “functional verification” and clarified in the text that our current work is primarily **phenotypic and depletion-based**. We now emphasize that future studies will include cross-presentation assays (e.g., using OVA models) and T-cell co-cultures to validate APC functionality. These points are now explicitly stated in the Discussion and Limitations sections **(Pages 14-15).**

**Comment 2:**

Furthermore, all experiments are conducted only in 7-8 weeks old mice, and do not explore the age-related variations in the frequencies and distributions of these cDC subsets. Given the plasticity of the splenic microenvironment with age, what are the chances that the patterns may be age-specific?

**Response:**

This is a valid point. We have added a statement in the **Discussion/Limitations section (Page 14)** acknowledging that age-related dynamics of these subsets remain to be investigated and represent an important future direction.

**Comment 3:**

Do these different cDC subsets have different spatial distributions within the spleen?

**Response:**

We agree that spatial context is critical. While our current study did not include spatial transcriptomics or immunohistochemistry, we have now explicitly mentioned in the **Discussion/Limitations section (Page 14)** that **spatial localization** of these subsets will be a key focus of our future work.

**Comment 4:**

The assumptions that certain cDC subsets are resident or migratory are completely based on their immunophenotypes, without sufficient definitive proof. Authors should adopt requisite validatory approaches by using cell-labeling and tracking.

**Response:**

We acknowledge this limitation. In the revised manuscript, we have rephrased our conclusions to indicate that residency or migratory potential is **inferred from marker expression and CL sensitivity**, and we note that definitive tracking experiments are needed in future studies. This is stated in the **Discussion/Limitations section (Page 14).**

**Comment 5:**

Do these different cDC subsets have any ontogenic relation among them? Authors might consider rechecking the flow cytometry data from their clodronate experiment to check whether the depletion of any one of the subsets leads to a compensatory increase in any other subsets; or causes an increase in the expression of one or more of the immunophenotypic markers corresponding to the depleted subsets within the surviving subsets. This might give important insights into possible ontogenic relations among them.

**Response:**

We did not directly assess ontogenic relationships in this study. However, we have added a comment in the **Discussion/Limitations section (Page 14)** suggesting that lineage-tracing or fate-mapping studies would be valuable to explore developmental trajectories among these subsets.

**Comment 6:**

The exact amount of liposomes injected (in terms of mass or molarity, not volume) needs to be reported.

**Response:**

We have now included the concentration of clodronate liposomes used (5 mg/mL) and the total dose per mouse (1 mg) in the **Methods section (Section 2.5, Page 6).**

**Comment 7:**

Statistical tests done against each experiment need to be mentioned in the respective figure legends. Moreover, the specific post-hoc tests for ANOVA need to be mentioned.

**Response:**

We have updated all figure legends to specify the statistical test used and include individual p-values where applicable. Specific details are now provided in the legends for **Figures 1, 2, and 3**. Furthermore, we have comprehensively detailed our statistical approach in the new **Section 2.7 (Statistical analysis, Page 7).**

**Comment 8:**

Most of the gating schemes show an absence of a singlet gating via FSC-A vs FSC-H combinations. How do the authors ensure that no doublets are present in their samples during flow cytometric acquisition?

**Response:**

We sincerely thank the reviewer for raising this critical point. We fully agree on the importance of singlet gating for data accuracy.

Upon careful re-examination of our raw flow cytometry data files, we regrettably found that **FSC-H, FSC-W, or any other channel Height/Width parameters were not recorded** during data acquisition for this specific experiment, preventing us from performing standard singlet gating.

To maximize the assurance of analyzing a single-cell population, we implemented the following compensatory measures:

1. **Stringent FSC-A/SSC-A Gating:** We applied strict gating on forward scatter (FSC-A) and side scatter (SSC-A) to define typical lymphocyte and monocyte regions, effectively excluding most debris and apparent cell aggregates. The complete gating strategy is displayed in the relevant figures.
2. **Sample Dilution and Optimized Acquisition:** We ensured the cell suspension was adequately diluted and used the instrument-recommended low flow rate during acquisition to minimize the probability of doublet formation at the source.

We acknowledge this as a **methodological limitation**, which we have explicitly stated in the **Discussion/Limitations section (Page 14).** We commit to enabling and recording FSC-A/FSC-H (or FSC-W) parameters for standard singlet gating in all future flow cytometry experiments.

**Comment 9:**

On the graphical data corresponding to experimental replicates, individual values need to be plotted alongside the measures of central tendency and dispersion, to show the exact distribution of data points.

**Response:**

We have updated the bar graphs in **Figures 1 and 2** to include individual data points superimposed on the mean ± SD (or median with interquartile range, as appropriate), as recommended.

**Response to Reviewer #2**

**Comment 1:**

The identified CD169⁻CD24⁺CD64⁺CX3CR1⁺MERTK⁺DCIR2⁺ subset within F4/80inter-low cDC2s is correlated with the co-expression of CX3CR1 and MERTK on TZM and claimed for their functional role in apoptotic clearance, just with the cell surface-based markers, without any functional experimental proof under in vitro or ex vivo settings.

**Response:**

We agree that direct functional evidence is lacking. We have revised the text in **Section 3.1 (Page 8)** to clarify that the functional potential for apoptotic clearance is **inferred from marker expression (CX3CR1+MERTK+)** and CL resistance. We now explicitly state that **direct phagocytosis assays** will be performed in future work.

**Comment 2 & 3:**

The identified subset within F4/80inter-low cDC2s has been considered tissue resident, with the absence of cell migration-associated surface markers like CCR7; however, no experimental data can be observed in the manuscript.

Again, being a splenic residential cell (claimed by the authors), it is quite likely to get exposed to various inflammatory stimuli or inflammatory mediators, which may induce migration-associated surface markers involved in migration, if not, at least within the intra-splenic regions. Experimental validation of the same under inflammatory conditions will uncover its true tissue-resident status.

**Response:**

We have toned down the claim of “tissue residency.” In **Section 3.1 (Page 8)**, we now refer to these cells as a **“putative tissue-resident”** population. We also added a note in the **Discussion/Limitations and Future Directions section (Page 14)** that inflammatory models should be used in the future to test marker stability and migratory behavior.

**Comment 4:**

The authors claimed the F4/80ʰⁱᵍʰCD169⁺ subset as a novel resident APC, only with phenotypic characterisation without any valid functional experiments in this regard.

**Response:**

We have removed the term “functional verification.” In **Section 3.2 (Page 9)**, we now describe this population as a **phenotypically distinct APC-like subset.** We acknowledge that T-cell activation assays are needed to confirm APC function and have included this as a future direction in the **Discussion (Page 14-15).**

**Comment 5:**

F4/80ʰⁱᵍʰCD4⁺CD8α⁺ cDC2s role in Th-biasness should be validated experimentally.

**Response:**

We agree. We have revised the text in **Section 3.3 (Page 10)** to indicate that the suggested Th-bias is **speculative based on marker profiles** and that cytokine polarization assays will be necessary to confirm functional bias. This is also mentioned in the **Discussion/Future studies (Page 14).**

**Comment 6:**

The flow-cytometric gating strategy described in the manuscript figure didn’t ensure that the cells are in a living (7-AAD vs SSC plot missing) and singlet state (FSC-A vs FSC-H plot missing).

**Response:**

We thank the reviewer for these important observations regarding both viability and singlet status.

1. **Cell Viability:** In this experiment, viable leukocytes were enriched using **strict morphological FSC-A/SSC-A gating** to exclude debris and dead cells (which typically exhibit low FSC and aberrantly high SSC). While fluorescent viability dyes (e.g., 7-AAD) were not used, this morphological gating is an established method for live cell enrichment. We have described this strategy in the **Methods section (Section 2.6, Page 7).**
2. **Singlet State:** As detailed in our response to **Reviewer #1, Comment 8,** the FSC-H/W parameters necessary for standard singlet gating were not recorded. We have implemented compensatory measures (stringent FSC-A/SSC-A gating and optimized sample handling) and acknowledged this as a key methodological limitation in the **Discussion (Page 14).**

We acknowledge that not using a viability dye is a limitation. Future studies will incorporate both viability staining and standard singlet gating.

**Comment 7:**

All animals considered for the experiments were from a similar age group, that is 7–8 weeks old C57BL/6 mice. This prevents deciphering the percentage or fraction of designated macrophage-like cDC throughout the growth of mice and susceptibility or biasness towards a particular inflammatory outcome, along with increasing age.

**Response:**

As also noted by Reviewer #1 (Comment 2), we have acknowledged this limitation. We have added a statement in the **Discussion/Limitations section (Page 13-14)** that age-related variation will be explored in future studies.

We believe that the revised manuscript has been significantly improved in direct response to all comments. We hope it now meets the high standards of PLOS ONE for publication.

Thank you for your consideration.

Sincerely,

Chunqing Yang, Ph.D.

Corresponding Author

Institute of Mental Health, Jining Medical University
